# Mutual Authentication Protocol for D2D Communications in a Cloud-Based E-Health System

**DOI:** 10.3390/s20072072

**Published:** 2020-04-07

**Authors:** Ana Paula G. Lopes, Paulo R. L. Gondim

**Affiliations:** Electrical Engineering Department, University of Brasilia, Brasilia 70910-900, Brazil; anagolembiouski@aluno.unb.br

**Keywords:** authentication, device-to-device, m-health, security, IoT

## Abstract

The development of the Internet of Things (IoT) predicts several new applications, some of which are designed to be incorporated into e-health systems, and some technologies, like cloud computing and device-to-device communication (D2D), are promising for use in the support of resource-constrained devices employed in Mobile-health (m-health) and Telecare Medicine Information Systems (TMIS). In a scenario with billions of devices predicted for the IoT, it is essential to avoid performance and security problems, among others. Security is fundamental for the achievement of optimal performance regarding the sensibility of e-health shared data and, especially, the anonymity of patients and other entities, while it is also essential to consider the scarcity of bandwidth in wireless networks. This paper proposes a new mutual authentication protocol for m-health systems, which supports D2D communication, ensuring security and surpassing the performance and security of other authentication procedures reported in the literature.

## 1. Introduction

Among the several applications for the development of the Internet of Things (IoT), Electronic-health(e-health)/Mobile-health(m-health) aim at providing health services through information and communication technologies. Such applications include, for example, monitoring by sensors coupled to the body of patients and connected by a wireless body area network (WBAN), diagnosis and remote provisioning of health services to patients over public channels.

The assistance of cloud servers is an alternative to supplying the large demands of storage and processing generated by multiple medical service providers and increasing operational efficiency. In Telecare Medical Information Systems (TMIS), doctors and patients can be assisted by a cloud server; patients send a report containing the sensors’ measures to the cloud server and a doctor collects the data, provides diagnosis and finally sends a diagnosis report to the cloud server. Both data exchanges are performed through public channels.

Additionally, the use of cloud servers as auxiliaries to storage and processing in e-health/m-health/TMIS requires special attention, due to the high sensitivity of the information exchanged with the cloud server and the entities involved. Information from the sensor measurements report and patient diagnosis can be crucial for saving lives and must not be accessed or modified by possible attackers.

A good example is the anonymity of entities, since the user of those systems may not be interested in having their identity disclosed. The disclosure of a patient’s identity can leave it vulnerable to the action of attackers against his/her life, or the access to personal information. One of the requirements for proper functioning of e-health/m-health/TMIS and other systems for the IoT is reductions in both the consumption of computational and communication resources for energy saving and the congestion on communication channels, given a large number of new emerging devices. Therefore, computational costs must be reduced for the optimization of power resources.

On the other hand, device-to-device (D2D) communication, commonly implemented by ad hoc wireless networks, enables patients’ devices to connect directly to a medical entity to send health data collected by sensors and receive diagnoses faster than in the traditional way. The constant monitoring of patients and analyses of health reports are crucial for the avoidance of medical conditions, such as strokes and heart attacks, because the chances of a person being sick can be detected much faster.

D2D communication provides a direct connection of devices with or without the intervention of traditional network infrastructure (e.g., 3rd Generation Partnership Project (3GPP) standards). Therefore, the ability to connect devices can provide data offload through nearby devices directly, thus reducing problems, such as congestion and scarcity of spectrum, and expanding network coverage by enabling devices to relay their data. D2D communication is promising for 5G technology and IoT due to its adaptation to support small and resource-constrained devices predicted by those two technologies. However, security schemes for D2D communication are still in the initial development stages, which require more research and studies for their improvement and consolidation, in addition to authentication and key agreement protocols adapted to them.

D2D is suitable for e-health/m-health/TMIS since it can accelerate the transmission of data and provide a connection to devices located outside the coverage of 3GPP networks. This might be the key for the success of e-health/m-health/TMIS applications, because most data exchanged provide information on patients’ health, e.g., heartbeat, blood sugar, and pressure, which is sensitive to delays for saving lives. Moreover, since e-health/m-health/TMIS devices are mostly resource-constrained, they require adapted traditional authentication protocols that consider their limitations and avoid costly data exchanges and computations. Therefore, new authentication and key agreement protocols can be designed to fulfill such requirements, when used for e-health/m-health/TMIS, while being secure and light to not overload them.

### 1.1. Main Contributions

The main contributions of this manuscript involve:

(a) a new symmetric cryptography-based mutual authentication and key agreement protocol for cloud-based e-health/m-health/TMIS that is adapted to support D2D communications;
(b) the guarantee offered by the proposed protocol of several security properties (e.g., confidentiality and anonymity) and resistance to attacks, such as replay, denial-of-service, and man-in-the-middle;
(c) computational, communication and energy costs evaluation and comparison with other authentication protocols from the literature, which demonstrated that the scheme proposed provided the best results;
(d) semi-formal validation of the proposed protocol, using Automated Validation of Internet Security Protocols and Applications (AVISPA) [1].

### 1.2. Structure of the Paper

The remainder of this manuscript is organized as follows: Section 2 looks at m-health and D2D communication; Section 3 describes some related work; Section 4 introduces the protocol; Section 5 and Section 6 address security and performance analyses, respectively; Section 7 addresses formal validation of the proposed protocol; finally, Section 8 provides the conclusions and future work.

## 2. M-Health Environments and Device-to-Device (D2D) Communication

M-health environments have grown with the emergence of technologies such as the IoT. Many applications have been developed, thus enabling the sharing of health information in real time among doctors and specialists. Some m-health systems consider groups of sensors coupled to a patient’s body: they send measurements to a mobile device and information to a gateway that can route the patient’s information to the respective health center. Consequently, both the number of devices and corresponding volume of exchanged data have increased and required a new perspective on the communication of small devices, especially when the scarcity of bandwidth is considered, which imposes reductions in the demand for radio resources. High-quality healthcare services, such as remote monitoring, mobile telemedicine, remote disease diagnosis, and emergency care, require the assurance of security of both system and communication channels through which messages are exchanged.

Since m-health aims to provide the monitoring and evaluation of vital signs and other important health information on patients and emergency situations 24 h a day and seven days a week, an important alternative is to use D2D communication for directly connecting devices, while maintaining control of resource allocation by the cellular network.

D2D communication refers to a direct communication between two mobile devices, i.e., communication that passes no cellular base station (BS) or network core [2], thus offering multiple services based on the proximity of the devices. The benefits of D2D communications regarding the requirements imposed by the 5G networks include higher data rate, low delay, coverage extension, reliability in communications, reduction of demands imposed over cellular networks by the use of traffic offloading, and instantaneous communications between devices [3]. Moreover, D2D communications can increase the spectral efficiency of the network and potentially improve throughput and energy efficiency [2].

Different levels of control and management of D2D communication are required by the operator. This operator has either no control, or full or partial control over the resource allocation among source, destination, and relay devices. According to the functionalities associated with the cellular infrastructure, the following types of D2D communications can be defined (Bastos and Cecílio Da [4], Llerena, Y.P. [5]):

- D2D communication with the establishment of an operator-controlled connection: source and destination devices talk and exchange data with each other without a BS; however, they are served by a BS to establish the link.
- D2D communication with the establishment of a device-controlled connection: source and destination devices communicate directly with each other with no operator control and must implement methods that guarantee limited interference with other devices on both the same layer and macrocell layer.

In this study, we have considered D2D communication with establishing/managing connection through the cellular infrastructure. Among its advantages are possible offloading between the mobile network and the D2D network, and efficient allocation of the spectrum if inband D2D is used [5]. Therefore, if some information collected by a sensor used by a patient is to be transmitted through a cellular network and indicates an emergency which demands rapid decision and actuation by a physician and/or a health center, D2D communications are an excellent alternative for its rapid transmission.

**Figure 1:**
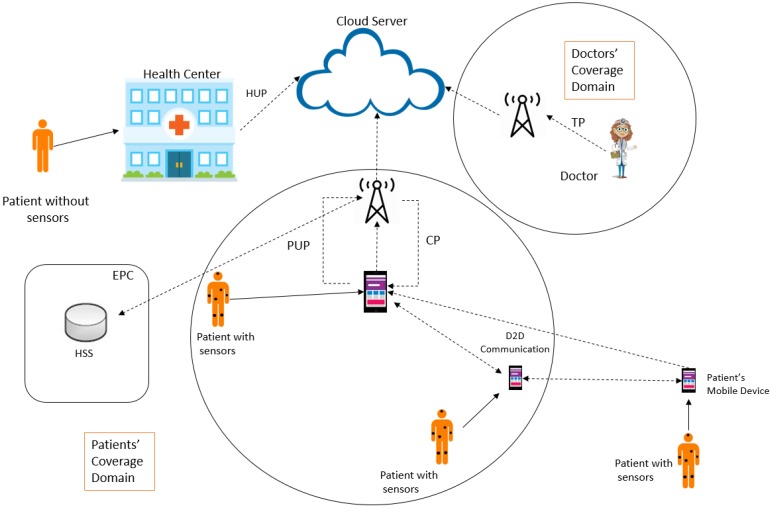
The architecture of the proposed scheme.

Moreover, regarding the protocol that uses D2D communications, D2D-based authentication protocols could be especially adapted to m-health architecture, thus reducing delays, dependency on 3GPP core network, and costs of message exchange. This issue has become more relevant because authentication protocols designed by traditional communication systems (e.g., 3GPP Evolved Packet System–Authentication and Key Agreement (EPS-AKA)) are not adequate for common requirements of 5G networks (e.g., higher data rate, low delay, low energy consumption and traffic offloading). Another important characteristic of D2D communication is possible coverage extension (see Figure 1, where a D2D-capable device outside the coverage area of the cellular network can be aided by another device inside that coverage area).

## 3. Related Work

Mohit et al. [6] and Chiou et al. [7] considered a cloud server an auxiliary entity that stores patients’ data, such as measures collected from sensors coupled to their bodies. Such data are encrypted and transmitted over public channels from the entities involved with the cloud server and vice versa, after the execution of mutual authentication and the generation of a session key. The authors designed protocols based on asymmetric and symmetric cryptography composed of four phases, namely health center upload (HUP), patient upload (PUP), treatment (TP) and checkup (CP). A security analysis that was conducted revealed some issues in the protocol of Chiou et al. [7]. According to Mohit et al. [6], it fails to preserve system anonymity and security if the patient’s device is lost or stolen. On the other hand, the protocol of Mohit et al. [5] fails to avoid a denial of service (DoS) attack. 

Jiang et al. [8] and Li et al. [9] also developed interesting approaches. Although the protocols considered no auxiliary cloud server (the entities authenticate themselves directly with the health center server through the Internet), they were developed for e-health/m-health/TMIS, similar to our protocol. The proposal of Li et al. [9] is based on asymmetric cryptography, whereas that designed by Jiang et al. [8] is based on symmetric cryptography. Both are composed of three phases in common, namely Initialization, Registration, and Authentication. Li et al. [9] accomplished all the security objectives considered in the security analysis section of this manuscript, while Jiang et al. [8] is vulnerable to the stealing of a patient’s device and involve confidentiality issues.

The protocols of Jiang et al. [10], Amin et al. [11] and Shen et al. [12] differ from those of Chiou et al. [7] and Mohit et al. [6] because they consider only the communication channel between the user (patient) and the cloud for security purposes. They also employ asymmetric cryptography based on the elliptic curves discrete logarithm problem (ECDLP) and comprise three phases, namely initialization, registration, and login/authentication. Jiang et al. [10] and Amin et al. [11] accomplished all the security objectives analyzed in this study; however, the protocol of Shen et al. [12] shows some security issues, such as lack of confidentiality and vulnerability to patient trackabillity due to loss/stealing of the patient’s mobile device. Finally, the protocols of [6,7,8,9,10,11,12] would better attend m-health requirements if D2D communication was used, because they would require less processing time, use of the device’s energy and band usage, reflecting in the reduction of costs.

Gunes et al. [13] proposed a hybrid model for Long Term Evolution (LTE) network-assisted D2D discovery and communication towards the integration of D2D into the 3GPP LTE architecture through the development of a device’s direct discovery model and optimization of the establishment of communications. It is based on the Proximity Services (ProSe) standard developed by 3GPP and its security requirements for D2D communication. However, it does not consider m-health adapted architecture and, consequently, would need to receive modifications to guarantee the security necessary for m-health.

Zhang et al. [14] developed an m-health authentication scheme for D2D communication. Based on the ECDLP, it is a certificateless signcryption scheme (CLGSC) that considers the necessity of protecting data from eavesdropping on the relays involved in D2D communication. However, it differs from ours because it does not consider a cloud server an auxiliary in the scheme.

Our protocol uses D2D communication for e-health/m-health/TMIS for enabling the transmission of large amounts of data, such as health reports with images, sound, and video, between devices in a short-range. It can accomplish a high data rate and lower energy consumption in comparison with traditional access technologies (e.g., 3GPP LTE, according to Kar and Sanyal [15]).

The literature reports some authentication and key agreement protocols for D2D communication; however, they are not designed for m-health environments. For example, Wang and Yan [16] developed two authentication protocols for D2D, one based on the hash message authentication code (HMAC) and the other based on identity-based signatures (IBS). Hsu et al. [17] proposed a group authentication protocol for D2D based on identity-based encryption (IBE) and Diffie Hellman key exchange (DHKE).

In terms of standardization activities, 3GPP has started to standardize D2D communication for its network architecture and developed technical reports and technical specifications (TS) (e.g., TS 33.303 [18], TS 23.303 [19] and TR 36.843 [20]), which describe security aspects, device discovery and, configuration for D2D communication.

Table 1 shows a comparison among some studies relevant to the design of our protocol.

## 4. Proposed Protocol

The proposed protocol is based on challenge-response and was developed as a secure and efficient mutual authentication scheme alternative, without incurring high computational and communication costs. The use of symmetric cryptography may generate security issues due to key exchanges over public channels. However, the proposed protocol does not exchange keys or real identities over insecure channels, as explained in Section 5.4 and Section 5.7, and consequently, it is not affected by these problems. We also propose a D2D communication environment, which provides the chance for devices inside the 3GPP network to do data offloading and the connection to devices outside the coverage area to be connected and send their owner’s health reports.

Figure 1 shows the system architecture, which is composed of a health center, a cloud server, patients with and without sensors, patient’s devices, doctors, the 3GPP access technology, the evolved node B (eNB) and the 3GPP evolved packet core (EPC), represented by the home subscriber server (HSS). There are two coverage domains. The first is the device’s coverage domain, which is composed of devices located inside the 3GPP coverage area and of devices located outside the coverage area. Patients located outside the coverage area can access the 3GPP network relaying their data through devices located inside the coverage area. The other domain is the 3GPP domain where the doctor is located.

Patients without sensors visit the health center in order to collect identity information to be used in future mutual authentication sessions. The health center must perform mutual authentication with the cloud server prior to sending its patients’ data. Patients’ devices perform mutual authentication with the cloud server prior to sending the data collected from the respective sensors. Devices with direct access to the 3GPP infrastructure might use it to reach the cloud server. Those devices located outside of the coverage area can execute the mutual authentication using D2D communication, prior to sending health reports. In the second case, the other D2D devices in the path to the cloud server are used as relays. A device with a direct connection with 3GPP infrastructure might choose to send its information through D2D communication to perform data offload, which is not addressed in this work.

Finally, each doctor also has to execute mutual authentication to obtain patients’ reports, evaluate their health conditions and guide them to the more suitable treatment.

The following sections detail each of the phases necessary to perform the mutual authentication of the entities considered and the cloud server. The phases are the following: registration, health center upload, patient upload, treatment and checkup. Table 2 presents the notations used in the protocol.

### 4.1. Device Discovery Scheme

In order to undertake D2D communication, the devices must execute a device discovery to detect and identify devices in proximity [13]. The 3GPP technical specification TS 23303 [20] describes two models of devices discovered with no permission necessary from the User Equipment (UE) to be discovered or with authorization required. The first model is Model A “I am here”, in which devices broadcast some information to announce their existence and monitor if interested devices also shared their information. In the second model, Model B “Who is there?”, devices work as discoverers by broadcasting the characteristics which it expects to find in the nearby devices and waiting for the response of those which are eligible to fulfill its expectations.

In this work we adopt Model A. The device discovery is executed following the solution presented in Gunes et al. [13] and the technical specification TS 23303 [20], which is described as follows.

First, as mentioned by [13], each device must prove to be authentic to the HSS, which checks if the International Mobile Subscriber Identity (*IMSI*) of the device matches the identity of the device registered in the database and if that device is eligible to perform D2D. If the verification passes, the devices are enabled to perform D2D communication. The authorization is stored in the eNB and refreshed at the expiration of a validity timer.

Next, it adopts a model with direct discovery among devices through a dedicated ProSe server, one of the solutions presented by [13] and based on the specifications of TS 23303 [20]. The devices detect and identify each other using Evolved Terrestrial Radio Access Network (E-UTRAN) or Wireless Local Area Network (WLAN) direct radio signals to share their identities.

### 4.2. Registration Phase

This phase is performed to provide the exchange of important parameters used in the next phases, which regards authentication. Each device must have its *IMSI* registered in the HSS. This registration occurs offline and is executed by the manufacturer.

Next, it is necessary to register the health center, patients and doctors in the cloud server. This registration occurs through a secure channel. Each entity generates *k* different random numbers *R_k_* and calculates a set of temporary identities, *TID_x_ = h_1_(ID_x_ || R_k_)*, which are individually used at each authentication session initiated by the entities. The use of real identities associated with a random number in the calculation of temporary identities guarantees its uniqueness. They send their real identity *ID_x_*, and temporary identities *TID_x_* to the cloud server, which stores the data to be used in the following phases. If all temporary identities of a certain entity are used, a new registration phase is performed. If a real identity is revoked, it is necessary to perform a special registration phase, indicating which was the identity revoked and the new equivalent identity. Only registered entities can perform the following phases.

### 4.3. Health Center Upload Phase (HUP)

**Figure 2:**
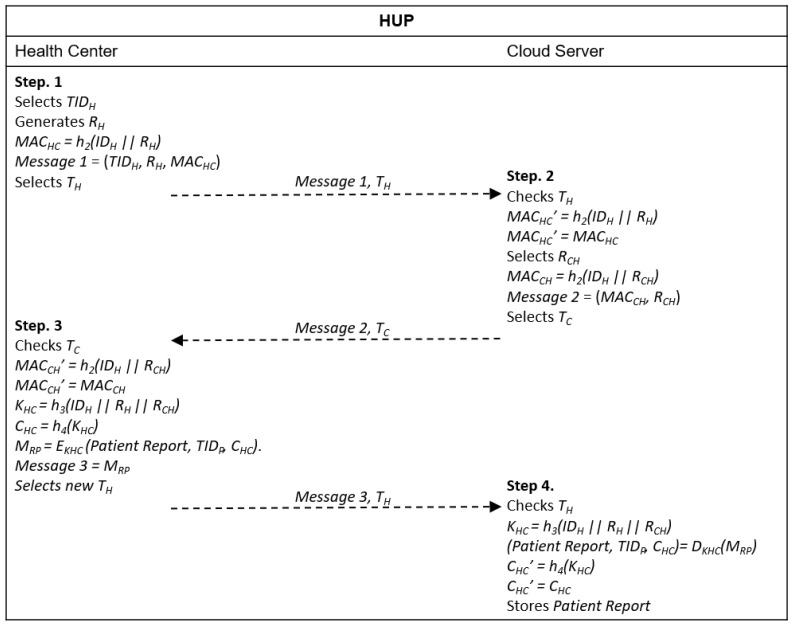
Message exchange in health center upload (HUP).

It is considered an insecure channel for this phase. Its aim is the mutual authentication among entities to allow secure transmission of the patient’s collected data, from the health center to the cloud server. The complete procedure is shown in Figure 2. The HUP phase starts when the user goes to the health center for a health inspection and receives a login and a password to access the patient’s system in its mobile device. The patient can access his/her health information whenever wanted by inserting the login/password pair on his/her device.

Step 1. The health center selects a *TID_H_* and generates a random number *R_H_*. Then, it calculates *MAC_HC_ = h_2_(ID_H_ || R_H_)* and sends *Message 1* = (*TID_H_*, *R_H_*, *MAC_HC_*) to the cloud server with a timestamp *T_H_*.

Step 2. After receiving *Message 1* and *T_H_* from the health center, the cloud server verifies if *T_H_* is valid. If the verification fails, the procedure is terminated. Otherwise, the cloud server calculates *MAC_HC_’ = h_2_(ID_H_ || R_H_)* using the real identity of the health center received in the registration phase and the random number received in *Message 1*. It then verifies if *MAC_HC_’ = MAC_HC_*. If the verification fails, the procedure ends because an intruder has been detected. Otherwise, the cloud server authenticates the health center, selects a random number *R_CH_*, calculates *MAC_CH_ = h_2_(ID_H_ || R_CH_)* and sends *Message 2* = (*MAC_CH_, R_CH_*) with a timestamp *T_C_* to the health center.

Step 3. The health center receives *Message 2* and *T_C_* from the cloud server and checks if timestamp *T_C_* is valid. If the validation fails, the procedure ends. Otherwise, the health center calculates *MAC_CH_’ = h_2_(ID_H_ || R_CH_)* and verifies if *MAC_CH_’ = MAC_CH_*. If the verification fails, the procedure is terminated because an intruder has been detected. Otherwise, the health center authenticates the cloud server and generates the session key, *K_HC_ = h_3_(ID_H_ || R_H_ || R_CH_)* and the session key validator, *C_HC_ = h_4_(K_HC_)*. It then uses the session key to encrypt the patient’s report, *M_RP_ = E_KHC_ (Patient Report, TID_P_, C_HC_)* and finally sends *Message 3 = M_RP_* and a new timestamp *T_H_* to the cloud server. 

Step 4. The cloud server receives {Message 3, *T_H_*} and verifies *T_H_*. If the verification fails, it terminates the procedure. Otherwise, it calculates the session key *K_HC_ = h_3_(ID_H_ || R_H_ || R_CH_)* and decrypts the patient’s report, *(Patient Report, TID_P_, C_HC_) = D_KHC_(M_RP_)*. It then calculates *C_HC_ = h_4_(K_HC_)* and verifies if *C_HC_’ = C_HC_*. If the verification fails, it ends the procedure. Finally, the cloud server stores the patient´s report with the respective identities.

### 4.4. Patient Upload Phase (PUP)

**Figure 3:**
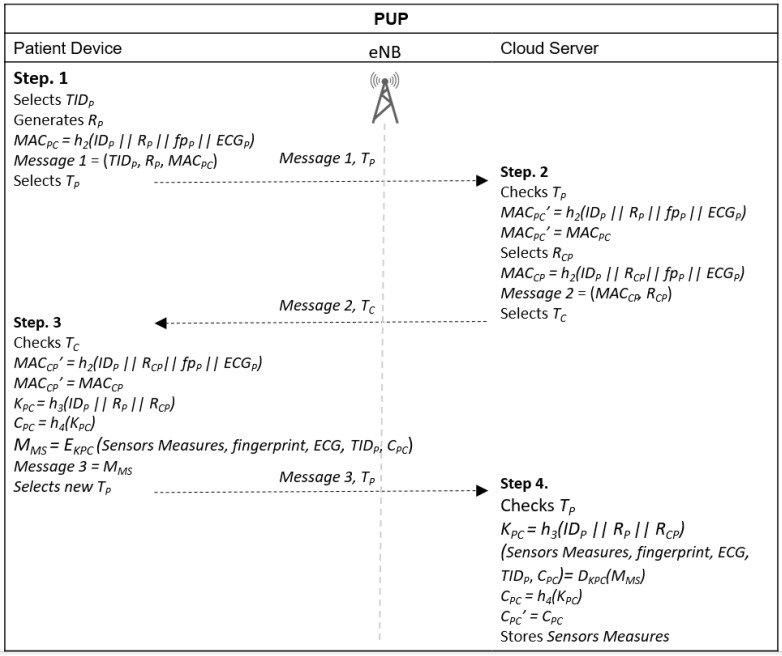
Message exchange in patient upload (PUP) for direct access to 3GPP infrastructure.

**Figure 4:**
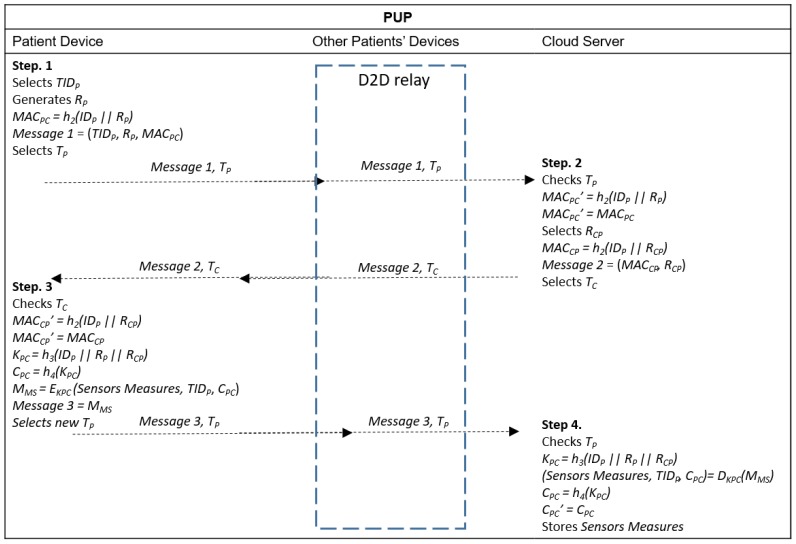
Message exchange in PUP when device-to-device (D2D) communication is adopted to reach the 3GPP infrastructure and the cloud server.

The PUP phase is performed over an insecure channel. The focus of PUP is the mutual authentication between patients and the cloud server and the generation of a session key to encrypt health information measured by the sensors attached to the user’s body, prior to sending it to the cloud server. The complete procedure is shown in Figure 3 and Figure 4. The PUP phase starts when the patient’s device requests, from the sensors attached to the user’s body, the health information measured and collected and stores them.

If necessary, the device discovery is performed in order to find other nearby devices as detailed in Section 4.1, based on proximity. However, first, they need to authenticate with a 3GPP network to prove their reliability. All devices interested in performing D2D communication selects a random number *R_P_*, calculates and sends the hash of its IMSI to the HSS to be authenticated:

*Auth_p_* = *h_1_(IMSI_p_ || R_P_)*

The HSS receives each *Auth_p_*, calculates *Auth’_p_* = *h_1_(IMSI_p_ || R_P_)* and verifies if *Auth_p_ = Auth’_p_*. If the verification passes, it authenticates the device. All devices authenticated by the HSS are enabled to perform D2D.

Then, the devices interested in D2D broadcast their TID_Di-j_ to reach other devices nearby, which might demonstrate the intention of establishing a connection with them. Next, they send their own temporary identities to signalize their existence and position. Now, a device located outside the coverage area or inside the coverage area but without access to the 3GPP network can perform their authentication with the cloud server by relaying their messages through the nearby devices, until the 3GPP network is reached.

Step 1. Calculates *MAC_PC_ = h_2_(ID_P_ || R_P_ )* and sends *Message 1* = (*TID_P_*, *R_P_*, *MAC_PC_*) with a timestamp *T_P_* to the cloud server. A device with direct access to the 3GPP network can choose to send data directly or to perform offload through D2D communication until the cloud server is reached. Devices without the 3GPP coverage send their data through D2D communication.

Step 2. The cloud server receives *Message 1* and *T_P_* and verifies if *T_P_* is valid. If the verification fails, the procedure is terminated. Otherwise, it calculates *MAC_PC_’ = h_2_(ID_P_ || R_P_ )* and verifies if *MAC_PC_’ = MAC_PC_*. If the verification fails, the procedure is interrupted. Otherwise, the cloud server authenticates the device, selects a random number *R_CP_*, calculates *MAC_CP_ = h_2_(ID_P_ || R_CP_)* and sends *Message 2* = (*MAC_CP_, R_CP_*) with a timestamp *T_C_* to the patient. 

Step 3. After receiving *Message 2* and *T_C_* from the cloud server, the patient checks if *T_C_* is valid. If the validation fails, the procedure ends. Otherwise, it calculates *MAC_CP_’ = h_2_(ID_P_ || R_CP_)* and verifies if *MAC_CP_’ = MAC_CP_*. If the verification fails, the procedure is terminated. Otherwise, the patient authenticates the cloud server, generates the session key *K_PC_ = h_3_(ID_P_ || R_P_ || R_CP_)* and calculates *C_PC_ = h_4_(K_PC_)*. He/she then encrypts the sensors measures using the session key, *M_MS_ = E_KPC_ (Sensors Measures, TID_P_, C_PC_)* and sends *Message 3 = M_MS_* with a new timestamp *T_P_* to the cloud server.

Step 4. The cloud server receives {*Message 3*, *T_P_*} and verifies if *T_P_* is valid. If the verification fails, it terminates the procedure. Otherwise, it calculates the session key *K_PC_ = h_3_(ID_P_ || R_P_ || R_CP_)*, decrypts the sensors measures, (*Sensors Measures, TID_P_, C_PC_) = D_KPC_(M_MS_*), calculates *C_PC_ = h_4_(K_CP_)* and verifies if *C_PC_’ = C_PC_*. If the verification fails, it terminates the procedure. Otherwise, the cloud server stores the sensors’ measures with the respective identities.

### 4.5. Treatment Phase (TP)

**Figure 5:**
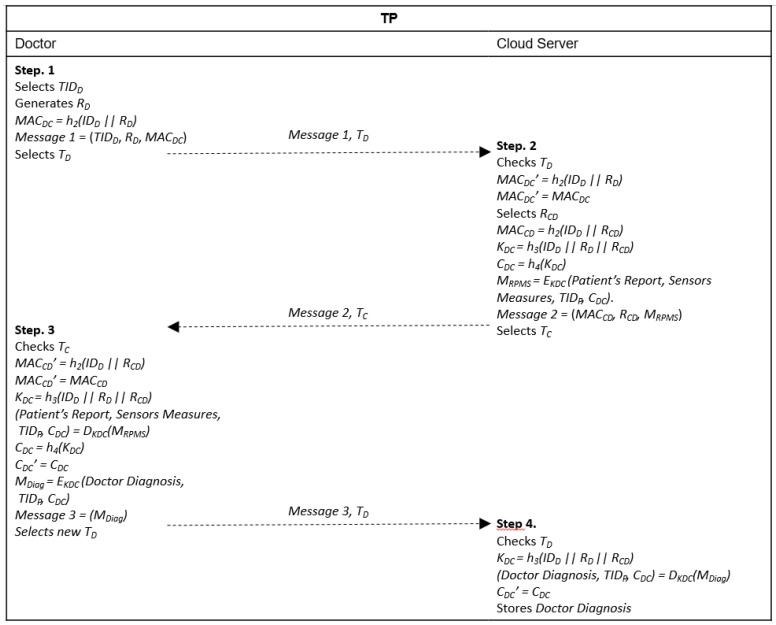
Message exchange in treatment phase (TP).

This phase is performed over an insecure channel. It aims at mutual authentication between the doctor and the cloud server and generation of a session key for encrypting the patient’s health report and sensors’ measures before they are sent to the doctor, in addition to encrypting the doctor’s diagnosis before it is sent to the cloud server. The complete procedure is shown in Figure 5.

Step 1. The doctor selects one of his/her temporary identities *TID_D_*, generates a random number *R_D_*, calculates *MAC_DC_ = h_2_(ID_D_ || R_D_)* and sends *Message 1* = (*TID_D_*, *R_D_*, *MAC_DC_*) with a timestamp *T_D_* to the cloud server.

Step 2. The cloud server receives {*Message* 1, *T_D_*} and verifies if *T_D_* is valid. If the verification fails, the procedure is terminated. Otherwise, it calculates *MAC_DC_’ = h_2_(ID_D_ || R_D_)* and verifies if *MAC_DC_’ = MAC_DC_*. If the verification fails, the procedure is interrupted. Otherwise, the cloud server authenticates the doctor, selects a random number *R_CD_* and calculates *MAC_CD_ = h_2_(ID_D_ || R_CD_)*, a session key *K_DC_ = h_3_(ID_D_ || R_D_ || R_CD_)* and *C_DC_ = h_4_(K_DC_)*. It then uses the doctor’s real identity to obtain the patient´s report and sensors’ health information measures previously stored in the cloud and prepare the information to be sent to the doctor, encrypting the data with the session key calculated, *M_RPMS_ = E_KHC_ (Patient Report, Sensors Measures, TID_P_, C_DC_)*. Finally, it sends *Message 2* = (*MAC_CD_, R_CD_, M_RPMS_*) with a timestamp *T_C_* to the doctor.

Step 3. The doctor receives {*Message* 2, *T_C_*} and checks if *T_C_* is valid. If the validation fails, the procedure ends. Otherwise, the health center calculates *MAC_CD_’ = h_2_(ID_D_ || R_CD_)* and verifies if *MAC_CD_’ = MAC_CD_*. If the verification fails, the procedure is terminated. Otherwise, the doctor authenticates the cloud server, generates the session key *K_DC_ = h_3_(ID_D_ || R_D_ || R_CD_)*, decrypts *M_RPMS_* to obtain the patient’s report and the health information measured by the sensors, *(Patient’s Report, Sensors Measures, TID_P,_ C_DC_) = D_KDC_(M_RPMS_)*, calculates *C_DC_’ = h_4_(K_DC_)* and verifies if *C_DC_’ = C_DC_*. Then, he/she analyzes the data received, generates the patient’s diagnosis, encrypts it, *M_Diag_ = E_KDC_ (Doctor Diagnosis, TID_P_)* and finally sends *Message 3 = M_Diag_* and a new timestamp *T_D_* to the cloud server.

Step 4. After receiving *Message 3* and *T_D_*, the cloud server verifies if *T_D_* is valid. If the verification fails, it terminates the procedure. Otherwise, it calculates the session key *K_DC_ = h_3_(ID_D_ || R_D_ || R_CD_)*, *C_DC_’ = h_4_(K_DC_)* and verifies if *C_DC_’ = C_DC_*. If the verification fails, it interrupts the procedure because the message was not originated from the authenticated doctor and might have been forged by an intruder. If the verification succeeds, the cloud server uses the session key to decrypt the doctor’s diagnosis and its respective temporary identity, *(Doctor Diagnosis, TID_D_) = D_KDC_(M_Diag_)*. Finally, it stores the doctor’s diagnosis with its respective identities.

### 4.6. Checkup Phase (CP)

**Figure 6:**
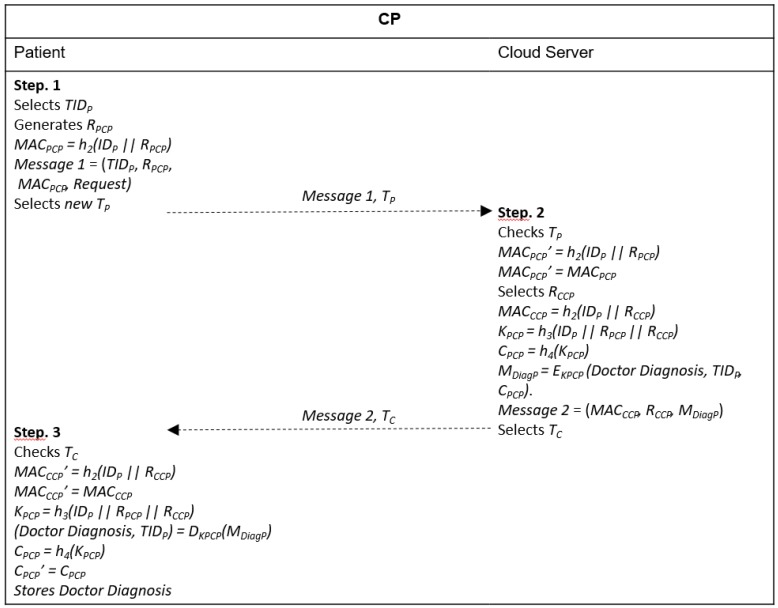
Message exchange in checkup phase (CP).

This phase is performed over an insecure channel and aims at a new mutual authentication between the patient and the cloud server and generation of a new session key for encrypting the doctor’s diagnosis before the cloud sends it to the patient. The complete procedure is shown in Figure 6.

Step 1. The patient generates a new random number *R_PCP_*, calculates *MAC_PCP_ = h_2_(ID_P_ || R_PCP_)* and sends *Message 1* = (*TID_P_*, *R_PCP_*, *MAC_PCP_, Request*) with a timestamp *T_P_* to the cloud server. Devices with direct access to the 3GPP network can send their data directly or use D2D communication to reach the cloud server. Devices without the 3GPP coverage must send their data through D2D communication.

Step 2. After receiving *Message* 1 and *T_P_*, the cloud server verifies if *T_P_* is valid. If the verification fails, the procedure is terminated. Otherwise, it calculates *MAC_PCP_’ = h_2_(ID_P_ || R_PCP_)* and verifies if *MAC_PCP_’ = MAC_PCP_*. If the verification fails, the procedure ends. Otherwise, it authenticates the patient, selects a random number *R_CCP_*, calculates *MAC_CCP_ = h_2_(ID_P_ || R_CCP_)*, generates the session key *K_PCP_ = h_3_(ID_P_ || R_PCP_ || R_CCP_)* and computes *C_PCP_ = h_4_(K_PCP_)*. It then uses the session key to encrypt the doctor’s diagnosis, *M_DiagP_ = E_KPCP_ (Doctor’s Diagnosis, TID_P_, C_PCP_)* and sends it to the patient *Message 2* = (*MAC_CCP_, R_CCP_, M_DiagP_*) with a timestamp *T_C_*.

Step 3. The patient receives {*Message* 2, *T_C_*} and checks if *T_C_* is valid. If the validation fails, the procedure is terminated. Otherwise, he/she calculates *MAC_CCP_’ = h_2_(ID_P_ || R_CCP_)* and verifies if *MAC_CCP_’ = MAC_CCP_*. If the verification fails, the procedure is interrupted. Otherwise, he/she authenticates the cloud server, generates the session key *K_PCP_ = h_3_(ID_P_ || R_PCP_ || R_CCP_)*, decrypts the doctor’s diagnosis, *(Doctor’s Diagnosis, TID_P_, C_PCP_) = D_KPCP_(M_DiagP_)*, calculates *C_PCP_ = h_4_(K_PCP_)* and verifies if *C_PCP_’ = C_PCP_*. If the verification fails, it ends the procedure. Otherwise, the patient stores the doctor’s diagnosis and looks for a convenient treatment.

## 5. Security Analysis

This section presents the security objectives accomplished by the protocol. Table 3 shows a security comparison between the proposed protocol and those designed by Chiou et al. [7] and Mohit et al. [6].

### 5.1. Mutual Authentication

In our protocol, each entity calculates a MAC to perform mutual authentication with the cloud server and vice versa. For example, in the HUP phase, the health center calculates MAC_HC_ = h_2_(ID_H_ || R_H_ ) and sends it to the server cloud, which calculates MAC_HC_’ = h_2_(ID_H_ || R_H_) and verifies if MAC_HC_’ = MAC_HC_. If the verification is successful, the server cloud authenticates the health center, calculates its own MAC_CH_ = h_2_(ID_H_ || R_CH_) and sends it to the health center, which calculates MAC_CH_’–h_2_(ID_H_ || R_CH_) and verifies if MAC_CH_’ = MAC_CH_. If the verification succeeds, the health center authenticates the server cloud and the mutual authentication procedure is complete. A similar procedure is performed in the PUP, TP and CP phases.

### 5.2. Forward/Backward Secrecy

The forward and backward secrecies are guaranteed by the use of random values (R_H_, R_CH_, R_P_, R_CP_, R_D_, R_CD_, R_PC_, R_CPC_) newly generated in each authentication session, during the calculation of the system keys, as the one generated in the PUP phase K_CP_ = h_3_(ID_P_ || R_P_ || R_C_). Therefore, if an intruder discovers old system keys, it cannot use them in future authentication sessions (backward secrecy). On the other hand, if an intruder discovers future system keys, it cannot use them in past authentication sessions (forward secrecy).

### 5.3. Confidentiality

The system’s confidentiality is guaranteed by the access control of the patient’s mobile device. A possible user must insert a login and password to access his/her information in the system. Consequently, sensitive information is available only to authorized users. An authentication procedure is performed between the cloud and an entity in each phase for the generation of a session key that will encrypt the patient’s data before it is exchanged on a public channel.

### 5.4. Non-Repudiation

At the beginning of each phase in the protocol, the entities send the cloud their temporary identities (TID_H_, TID_P_, TID_D_) and a MAC calculated with their real identities (ID_H_, ID_P_, ID_D_). The cloud also sends to the entities a MAC containing their real identities. Since real identities are known only by the cloud and each respective entity, a valid MAC can be generated only by them. The session keys established among the cloud and the entities also depend on their real identity, therefore, neither the cloud nor the entities can deny the message they originated.

### 5.5. Anonymity

Anonymity is assured only by entities’ temporary identities (TID_H_, TID_P_, TID_D_), while messages are exchanged on an insecure channel during the authentication procedure, which protects their real identities. The identity of the cloud server is protected because it is not used in the authentication procedure, hence, not exchanged on an insecure channel.

### 5.6. Non-Traceability

The use of different temporary identities and newly generated random numbers in each new authentication session generates different parameters exchanged. Therefore, outsiders cannot track patients by the parameters exchanged on a public channel.

### 5.7. Session Key Security

Session keys are not exchanged on a public channel but securely calculated on each side involved in the authentication. Moreover, the security of the session keys established at each phase of the protocol is guaranteed through the use of entities’ real identities in the calculation, some secret information known only by the cloud server and the respective entities. For example, in HUP, the session key calculated is KHC = h_3_(IDH || RH || RCH); Consequently, an intruder cannot obtain or calculate a valid session key.

### 5.8. Patient’s Mobile Device Loss/Stealing 

The security objective is accomplished through the access control of the patient’s mobile device using a login and password. The system is only accessible if a valid login and password pair is inserted. If the mobile device is stolen or lost, no unauthorized person can access the patient’s system, because it would not have a valid login and password pair.

### 5.9. Impersonation Attack

The impersonation attack is avoided because neither the cloud server’s real identity nor the entities’ real identities are disclosed. Therefore, an attacker cannot impersonate them and generate a valid MAC, because its calculation depends on the entities’ real identities.

### 5.10. Replay Attack

The replay attack is avoided because all entities involved in our protocol use different random values freshly calculated in each authentication process. Therefore, an attacker cannot forge messages using old random values.

### 5.11. Denial of Service (DoS)

The prevention of this attack involves the inclusion of a verification parameter in each message exchanged in the authentication phases (HUP, PUP, TP, CP). The verification parameter used in our protocol was a timestamp and its validity was verified before the recipient processed each message. Therefore, if an attacker uses an invalid timestamp, the entire procedure is interrupted in time to prevent the DoS attack.

### 5.12. Man-in-the-Middle Attack

No intruder can perform a man-in-the-middle attack, because the session key cannot be forged with the use of only the parameters exchanged on the insecure communication channel. The session key calculation uses the entities’ real identities, which is a secret value not disclosed in the insecure channel.

Table 3 data are derived from the analysis of the data presented in [6] and [7]. The protocol designed by Chiou et al. [7] does not guarantee anonymity, non-traceability, and resistance to patient’s mobile device loss/stealing, which are three critical failures. First, as detected by Mohit et al. [6], in the protocol of Chiou et al. [7], the patient’s real identity is sent in plain text through a public channel, which compromises its anonymity. We observed it also affects the patient’s non-traceability. Second, as detected by Mohit et al. [6], the proposal of Chiou et al. [7] fails to be resistant to the patient’s mobile device loss/stealing, because it does not perform access control and requests login and password to the user, which makes the system vulnerable to the access of non-authorized people and hampers its confidentiality.

The protocol of Mohit et al. [6] fails to prevent DoS attack. No initial verification parameter is generated (timestamp, nonce, sequence number) to be sent with the parameters exchanged. Then, the validity of a simpler parameter is not verified before the recipient processes more complex calculations. Therefore, the protocol is vulnerable to DoS attacks, because the system of D2D devices is not robust enough to deal with message flooding. Our protocol accomplished all security objectives analyzed and can, therefore, be considered safer than those designed by Chiou et al. [7] and Mohit et al. [6].

## 6. Performance Analysis

This section addresses a performance analysis of our protocol and a comparison with those developed by Chiou et al. [7] and Mohit et al. [6]. The analysis evaluated computational, communication and energy costs and was based on the data available in [6] and [7], in parallel with their own cost evaluation. The registration phase of the protocol was not included in the analysis because it is performed over a secure channel and the focus of the comparisons was on operations executed and parameters exchanged over an insecure channel. It is considered that “n” is the number of devices executing mutual authentication with the cloud server using a traditional 3GPP network. It is also considered that “m” is the number of devices using D2D communication to perform mutual authentication with the cloud server.

### 6.1. Computational Cost

The execution time in seconds (s) of the operations considered is shown in Table 4. Chiou et al. [7] and Mohit et al. [6] adopted those values and performed tests with the following operational characteristics: CPU: Intel (R) Core (TM) 2 Quad Q8300, 2.50Hz; memory: 2GB; operational system: Windows 7 Professional.

All four phases were analyzed and all operations executed were considered. Table 5 shows a comparison of the computational costs among our protocol and those of Chiou et al. [7], Mohit et al. [6], details of the operations performed at each phase and the total time in seconds.

Our protocol required the lowest computational cost, therefore, it performs the operations necessary in a shorter time and offers the best computational cost, due to the exclusive use of symmetric cryptography (low communication cost) for the authentication procedures. Chiou et al. [7] and Mohit et al. [6] conducted some signature operations and bilinear pairing, which incurred higher computational costs.

**Figure 7:**
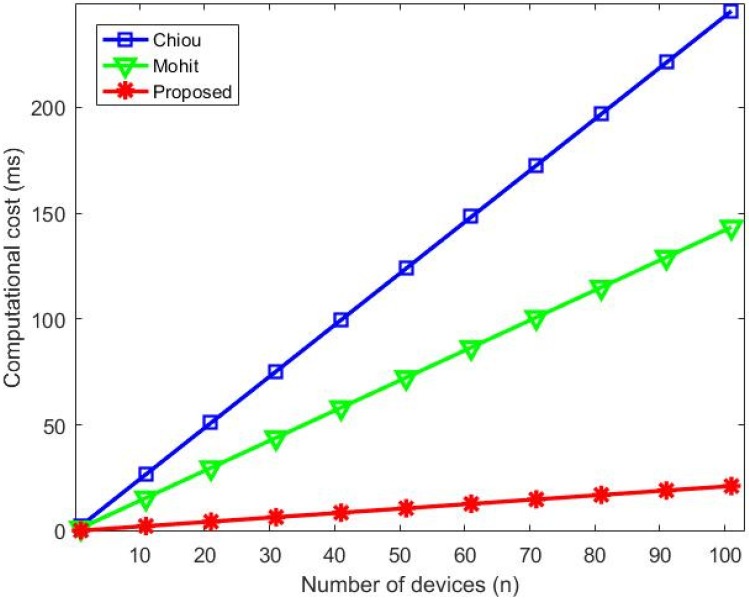
Computational cost comparison.

Figure 7 confirms with a graphic representation of costs that our proposed protocol has the best performance regarding computational costs when compared to [6] and [7].

### 6.2. Communication Cost

The evaluation of the communication costs considered messages exchanged over an insecure channel and parameters and their respective costs in bits (see Table 6).

The message exchange over an insecure channel was analyzed in each of the four common phases performed by our protocol and those of Chiou et al. [7] and Mohit et al. [6]. Table 7 shows comparisons of each phase and a comparison of the total communication cost of each protocol.

Our protocol required the lowest communication cost, hence, the best communication cost, due to the reduced number of parameters exchanged and choice of small parameters to be exchanged (identities, random numbers, timestamps) and the adaptation to D2D communication that offloads part of the traffic outside the 3GPP spectrum. The proposals of Chiou et al. [7] and Mohit et al. [6] required higher communication costs, because of the exchange of some costly signature parameters. Our protocol achieved the best performance, revealed by security and performance analyses.

**Figure 8:**
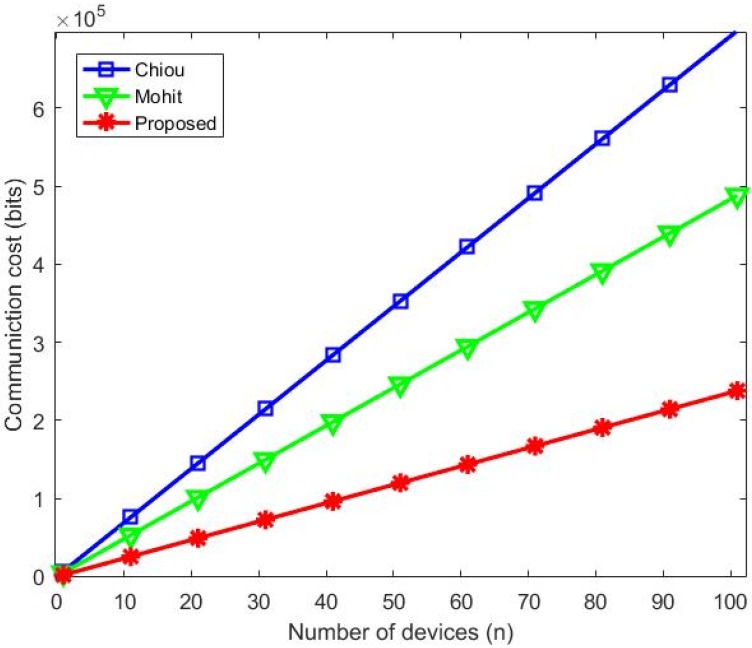
Communication cost comparison.

Figure 8 shows the best performance of the proposed protocol regarding communication costs. It considers that 30% of devices perform offload and use D2D communication to execute their mutual authentication. Reducing costs in the traditional 3GPP network.

### 6.3. Energy Cost

Kumar et al. [21] and He et al. [22] have proposed energy cost evaluation, which considers that the maximum CPU power of devices (W) is approximately 10.88 Watts. The energy overhead was calculated as follows: ETotal = CCTotal × W, where CCTotal is the computational cost calculated and presented in Section 5.1.

**Figure 9:**
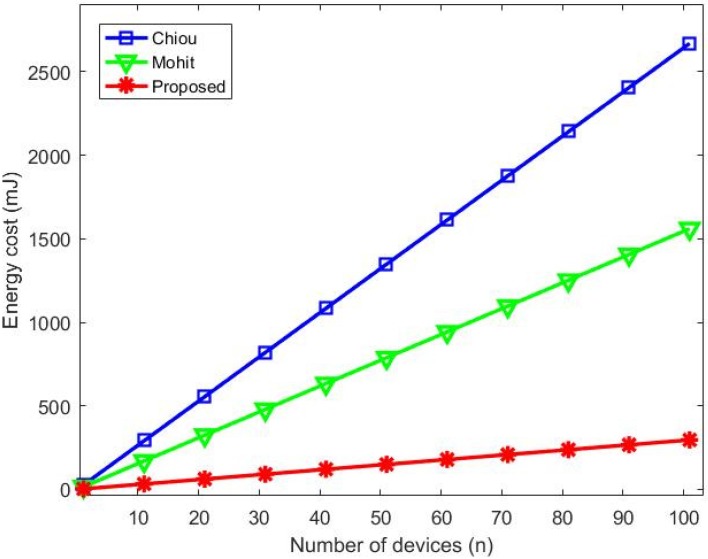
Energy cost comparison.

Table 8 and Figure 9 show the comparison of energy costs among our protocol and other protocols from [7] and [6]. Figure 9 shows that our proposed scheme has the best performance regarding energy. The energy cost directly related to the computational cost and consequently the graphic results are for both costs and are very similar.

Finally, the good results obtained in the security and performance evaluations prove that the proposed protocol can perform better than the works of [6] and [7]. Below are some aspects compared with the above-mentioned works:

(a) the protocols of Chiou et al. [7] and Mohit et al. [6] are based on asymmetric cryptography, while our approach is based on symmetric cryptography, which is able to produce lower computational and communication costs;
(b) the security flaws of Chiou et al. [7] and Mohit et al. [6] are avoided in our protocol using access control to the patient’s device, timestamps, temporary identities and freshly generated parameters in each authentication session;
(c) by contrast with our proposed scheme, the protocols of Chiou et al. [7] and Mohit et al. [6] do not support D2D communication, which is a promising technology for the development of e-health systems due to the agility of data transmission it can provide. The proposed protocol was designed thinking about the criticality of the health systems that in some cases might depend on the agility of data transmission to save lives.

## 7. Formal Validation

The proposed protocol was validated using Automated Validation of Internet Security Protocols and Applications (AVISPA) [1]. This is a semi-automated validation tool that permits the verification of security robustness of authentication protocols by checking the secrecy of key parameters and the vulnerability to intruders.

AVISPA validation is made through codes written in the high-level protocol specification language (HLPSL). The message exchange of the protocol is translated to HLPSL code, where each entity is defined as a communication agent that performs roles. Each entity has its own role defined, which contains all the parameters exchanged in the messages (States). All parameters that must remain secret are signalized and observed during the code execution. If no secret value is vulnerable to be discovered by intruders, the protocol is considered safe.

**Figure 10:**
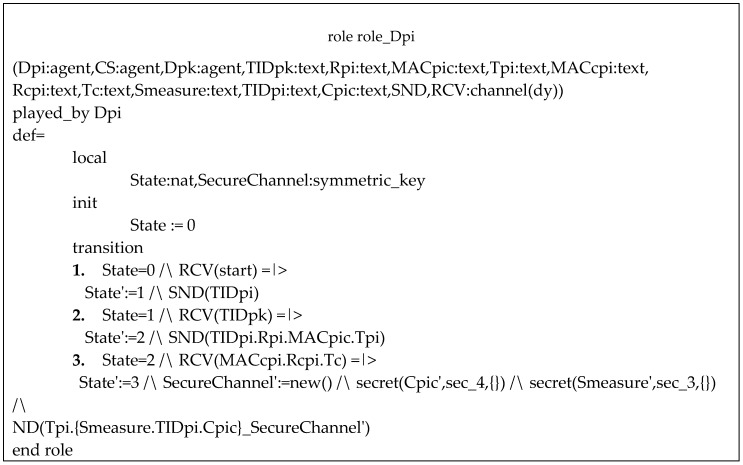
Role of D2D device Dpi in PUP phase.

**Figure 11:**
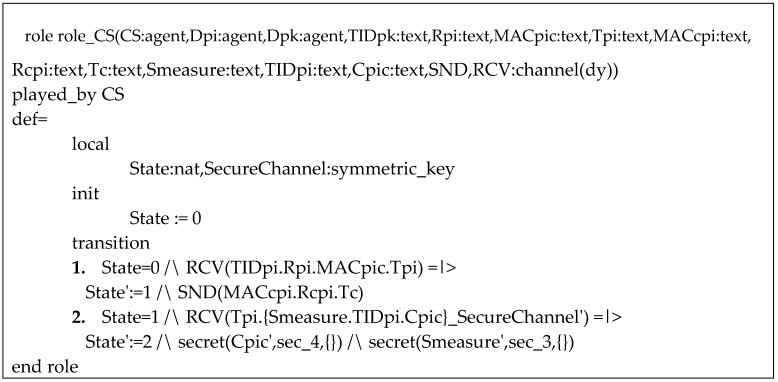
Role of the cloud server in PUP phase.

Our validation was made for each of the four phases performed over an insecure channel (HUP, PUP, TP and CP). Figure 10 presents the role for an ordinary device in the PUP phase, named Dpi in the code. Each State symbolizes the messages sent (SND) and received (RCV). Each parameter that must remain secret is signalized with a flag, such as sec_3 and sec_4 in Figure 10. The flag SecureChannel accompany an encrypted message and the parameters that are sent encrypted are signalized as secret (parameter). Figure 11 shows the role of the cloud server in PUP phase.

**Figure 12:**
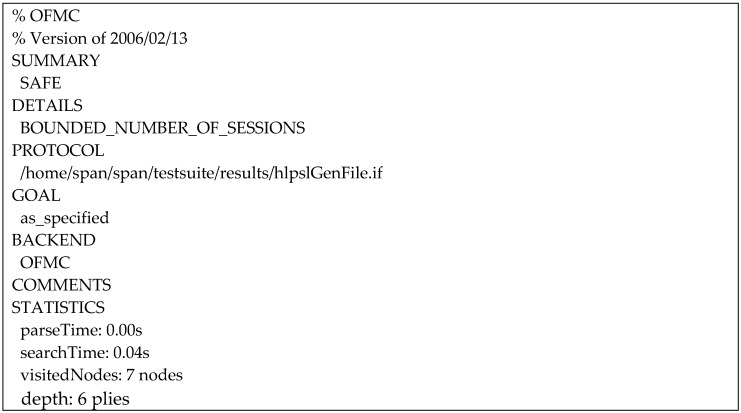
On-the-Fly-Model-checker (OFMC) analysis result.

We use two of the AVISPA’s four security evaluation backends in the validation of our proposed protocol: On-the-Fly-Model-checker (OFMC) [23] and Constraint Logic-based Attack Searcher (CL-AtSe) [24]. Figure 12 presents the results of the OFMC analysis of the PUP phase, which concluded that our proposed protocol is safe.

**Figure 13:**
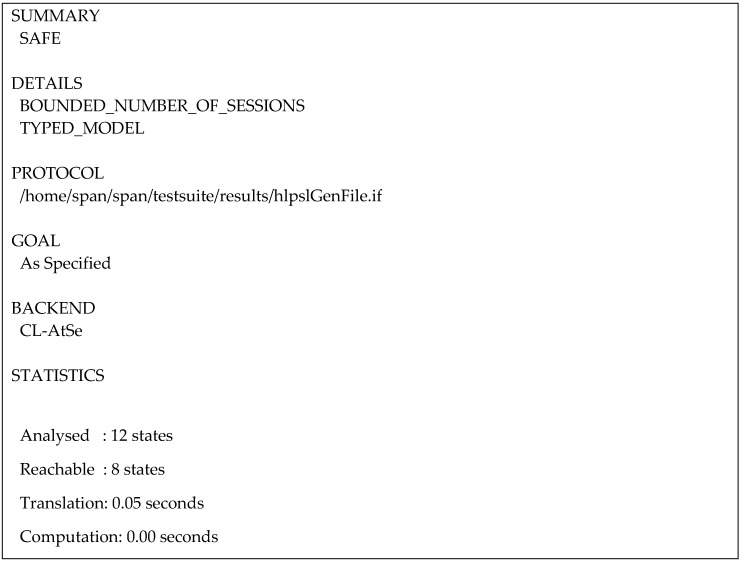
Constraint Logic-based Attack Searcher (CL-AtSe) analysis result for PUP phase.

Figure 13 shows the analysis of the PUP phase in the CL-AtSe backend and its respective results. Our proposed protocol was declared safe according to the analysis.

## 8. Conclusions

The application of e-health/m-health to the monitoring, diagnosis, and treatment of patients speeds up the provision of medical services. In many cases, the patient does not need to leave his/her home for a doctor’s appointment, which facilitates access to medical advice for patients with limited mobility, the elderly or patients located in hard access areas.

The protocols analyzed showed interest in the development of efficient and safe e-health/m-health/TMIS systems for protecting patients’ data and their respective identities. Our protocol showed itself to be suitable to e-health/m-health/TMIS and outperformed those of Chiou et al. [7] and Mohit et al. [6]. The protocol designed by Chiou et al. [7] does not control the access to patients’ mobile devices for avoiding their system’s exposure to intruders, if the device is lost or stolen, which is a problem with a simple solution. The protocol designed by Mohit et al. [6] fails to avoid the denial of service (DoS) attack. In addition, both protocols do not support D2D communication.

Furthermore, reductions in computational and communication costs are reinforced by using symmetric cryptography. Asymmetric cryptography demands more resource consumption than symmetric cryptography due to the execution of more complex operations such as elliptic curves [25]. Moreover, there are some common misconceptions such as to consider asymmetric cryptography safer than symmetric cryptography. In terms of cryptoanalysis, the length of the key and the computational work involved in breaking a cipher are essential for security evaluation. Symmetric cryptography is suitable to be used in situations in which costs must be reduced, such as for the resource-constrained devices used for m-health.

The performance and security analyses conducted confirmed that resource consumption could be reduced in our proposed solution when compared with other solutions that use asymmetric cryptography with no impact on the system’s security through the use of symmetric cryptography.

Future studies include storage cost analysis and comparison with related works and development of other mutual authentication protocols based on asymmetric cryptography for cloud-based e-health systems that accomplish more security objectives, as the objectives presented by Liu et al. [26], with reduced resource consumption.

The development of authentication and authorization protocols, considering CPS (cyber physical systems) ([27,28,29]) as well as security evaluation based on integrated systems of ambient-assisted living (AAL) and e-health (as in Rghioui et al. [30]) has also been considered. Finally, the influence of the mobility on the authentication of D2D communications ([31,32,33]) will be explored.

## Figures and Tables

**Table 1:** Comparison among some protocols.

	D2D Communication	m-Health/e-Health/TMIS	Type of Cryptography	Cloud Server
Chiou et al. [7]	No	Yes	Asymmetric	Yes
Mohit et al. [6]	No	Yes	Asymmetric	Yes
Jiang and Lian [8]	No	Yes	Symmetric	No
Li et al. [9]	No	Yes	Asymmetric	No
Wang and Yan [16]	Yes	No	Asymmetric	No
Hsu et al. [17]	Yes	No	Asymmetric	No
Zhang et al. [14]	Yes	Yes	Asymmetric	No
Our Protocol	Yes	Yes	Symmetric	Yes

**Table 2:** Notations used in the protocol.

Symbol	Description
x, y	Entities: patient (P), health center (H), doctor (D), cloud server (C).
*ID_x_/TID_x_*	Real identity of entity x/ Temporary identity of entity x.
*k*	Random numbers generated in the registration phase.
*R_k_*	k random number generated.
MACxy	Message Authentication Code generated from entity x to entity y.
*R_x_*	Random number generated by entity x.
*R_Cy_*	Random number generated by the cloud and sent to entity y.
*T_x_*	Timestamp generated by entity x.
*K_xy_*	Session key generated by entities x and y.
*C_xy_*	Validator of the session key generated by x and y.
*E_Kxy_/D_Kxy_*	Encryption/Decryption operation that used the session key generated by x and y.
*IMSI_x_*	International Mobile Subscriber Identity of device x
*h_1_*	Temporary identity generation hash function.
*h_2_*	MAC generation hash function.
*h_3_*	Session key generation hash function.
*h_4_*	Session key verifier generation hash function.
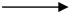	Secure channel.
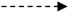	Insecure channel.

**Table 3:** Comparison of security objectives among protocols.

Security Objectives	Chiou et al. [7]	Mohit et al. [6]	Our Protocol
Mutual Authentication	Yes	Yes	Yes
Forward/Backward Secrecy	Yes	Yes	Yes
Confidentiality	No	Yes	Yes
Non-Repudiation	Yes	Yes	Yes
Anonymity	No	Yes	Yes
Patient’s Non-Traceability	No	Yes	Yes
Session Key Security	Yes	Yes	Yes
Resistance to patient’s mobile device loss/stealing	No	Yes	Yes
Resistance to Impersonation attack	Yes	Yes	Yes
Resistance to replay attack	Yes	Yes	Yes
Resistance to Denial of Service (DoS)	Yes	No	Yes
Resistance to man-in-the-middle attack	Yes	Yes	Yes

**Table 4:** Execution time of each operation considered.

Symbol	Description	Cost (Seconds)
T_S_	Execute/Verify a Signature	0.3317 s
T_P_	Bilinear Pairing	0.0621 s
T_E_	Encrypt/Decrypt (Symmetric)	0.0087 s
T_H_	One Way Hash Function	0.0005 s

**Table 5:** Computational cost of protocols.

	Chiou et al. [7]	Mohit et al. [6]	Proposed Protocol
HUP	nT_S_ + 3nT_P_ + 2nT_E_ + 7nT_H_	nT_S_ + 3nT_E_ + 11nT_H_	2nT_E_ + 8nT_H_
PUP	nT_S_ + 3nT_P_ + 2nT_E_ + 9nT_H_	2nT_S_ + 2nT_E_ + 10nT_H_	4nT_E_ + 9nT_H_
TP	2nT_S_ + 3nT_P_ + 2nT_E_ + 8nT_H_	2nT_S_ + 2nT_E_ + 9nT_H_	4nT_E_ + 8nT_H_
CP	nT_S_ + 2nT_P_ + 2nT_E_ + 8nT_H_	nT_S_ + 2nT_E_ + 5nT_H_	2nT_E_ + 8nT_H_
TOTAL (s)	5nT_S_ + 11nT_P_ + 8nT_E_ + 32nT_H_ = 2.43n	4nT_S_ + 9nT_E_ + 35nT_H_ = 1.42n	12nT_E_ + 33nT_H_ = 0.21n

**Table 6:** Cost of each parameter exchanged in bits.

Parameter	Cost
Random Number/Identity/Timestamp	48 bits
Bilinear Pairing/Hash	160 bits
Symmetric Key	128 bits
Signature (symmetric algorithm)	512 bits

**Table 7:** Comparison of communication costs in bits.

	Chiou et al. [7]	Mohit et al. [6]	Proposed Protocol
HUP	704n	592n	736n
PUP	1600n	1744n	736n + 736m + 208(m-1)
TP	2112n	1792n	864n
CP	1504n	1184n	736n
TOTAL	6920n bits	4832n bits	3072n bits

**Table 8:** Energy cost of protocols.

Reference/Protocol→	Chiou et al. [7]	Mohit et al. [6]	Proposed Protocol
TOTAL	(5nT_S_ + 11nT_P_ + 8nT_E_ + 32nT_H_) * 10.88 = 26.43n mJ	(4nT_S_ + 9nT_E_ + 35nT_H_) * 10.88 = 15.45n mJ	(12nT_E_ + 33nT_H_) * 10.88 = 2.93n mJ
